# Effect of An 84-bp Deletion of the Receptor-Binding Domain on the ACE2 Binding Affinity of the SARS-CoV-2 Spike Protein: An In Silico Analysis

**DOI:** 10.3390/genes12020194

**Published:** 2021-01-29

**Authors:** Gábor Kemenesi, Gábor Endre Tóth, Dávid Bajusz, György M. Keserű, Gabriella Terhes, Katalin Burián, Safia Zeghbib, Balázs A. Somogyi, Ferenc Jakab

**Affiliations:** 1 National Laboratory of Virology, Szentágothai Research Centre, University of Pécs, 7624 Pécs, Hungary; toth.gabor.endre@gmail.com (G.E.T.); zeghbib.safia@gmail.com (S.Z.); Somogyi.balazs.antal@gmail.com (B.A.S.); jakab.ferenc@pte.hu (F.J.); 2 Institute of Biology, Faculty of Sciences, University of Pécs, 7624 Pécs, Hungary; 3 Medicinal Chemistry Research Group, Research Centre for Natural Sciences, Magyar tudósok krt. 2, H1117 Budapest, Hungary; bajusz.david@ttk.hu (D.B.); keseru.gyorgy@ttk.hu (G.M.K.); 4 Department of Medical Microbiology and Immunobiology, University of Szeged, H6720 Szeged, Hungary; terhesga@gmail.com (G.T.); burian.katalin@med.u-szeged.hu (K.B.)

**Keywords:** selection, evolution, spike-mutant, deletion, attenuated, recessive

## Abstract

SARS-CoV-2 is a recently emerged, novel human coronavirus responsible for the currently ongoing COVID-19 pandemic. Recombination is a well-known evolutionary strategy of coronaviruses, which may frequently result in significant genetic alterations, such as deletions throughout the genome. In this study we identified a co-infection with two genetically different SARS-CoV-2 viruses within a single patient sample via amplicon-based next generation sequencing in Hungary. The recessive strain contained an 84 base pair deletion in the receptor binding domain of the Spike protein gene and was found to be gradually displaced by a dominant non-deleterious variant over-time. We have identified the region of the RBD that is affected by the mutation, created homology models of the RBDΔ84 mutant, and based on the available experimental data and calculations, we propose that the mutation has a deteriorating effect on the binding of RBD to the ACE2 receptor, which results in the negative selection of this variant. Extending the sequencing capacity toward the discovery of emerging recombinant or deleterious strains may facilitate the early recognition of novel strains with altered phenotypic attributes and understand key elements of Spike protein evolution. Such studies may greatly contribute to future therapeutic research and general understanding of genomic processes of the virus.

## 1. Introduction

Following the emergence of the novel SARS-CoV-2 virus in late 2019 in China [1], the first cases were confirmed in Europe in January from France and a recent report presented the first known emergence in December, Italy [2]. Nevertheless, the imported cases first evolved into epidemic situations in Lombardy region, Italy. Following the Italian outbreak, novel regions were seriously affected by the virus, mostly in Western European countries. During the first 11 months of the pandemic more than 60 million people have been infected, with 1.5 million fatalities [3].

SARS-CoV-2 is the seventh known human coronavirus and the third highly pathogenic human coronavirus species. HCoV-229E, -NL63, -OC43, and HKU1 are endemic human coronaviruses and known as common agents of mild upper and lower respiratory tract infections. Other human coronaviruses, the Middle Eastern Respiratory Syndrome (MERS), the Severe Acute Respiratory Syndrome viruses (SARS) and the recently emerged SARS-CoV-2 virus, can cause severe disease. In 2002–2003 there was an outbreak of viral pneumonia which affected at least 8000 individuals and the case fatality rate was approximately 10%. Since its emergence, SARS disappeared with no known natural infection to date. Whereas, in 2012, the MERS-CoV was fortuitously discovered in Saudi Arabia when a fatal human case of pneumonia occurred. The evolutionary origin was traced back to bats, whilst it is now widely established in dromedaries which are known as the intermediate host for its emergence. Sporadic spillover events from dromedaries to humans and further human-to-human transmission events are occurring to date [4].

The newly emerged *betacoronavirus*, namely SARS-CoV-2, belongs to the *sarbecovirus* subgenus along with SARS-CoV and bat SARS-like coronaviruses. Its +ssRNA genome length ranges between 29.8 kb to 29.9 kb. It has a typical CoV genome organization. With both capped 5′ end and polyadenylated 3′ end it acts directly as an mRNA. At the 5′ the orf1ab occupy more than the two third of the genome and encodes 16 nonstructural proteins, whereas, genes encoding the four structural proteins englobing surface (S), envelope (E), membrane (M), and nucleocapsid (N) proteins. In addition, six accessory proteins encoded respectively by the ORF3a, ORF6, ORF7a, ORF7b, and ORF8 genes are located at the 3′ end [5].

The surface of the virus is covered with the glycosylated Spike proteins (S), which mediate both virus binding and cell entry. After its attachment to the host cell receptor, namely angiotensin-converting enzyme 2 (ACE2), the S protein is cleaved to S1 and S2 by the host type 2 TM serine protease (TMPRSS2) and thus enhances the viral cell entry. It consists of 1273 aa and comprises a signal peptide at the N-terminal (aa 1–13) followed by the S1 (aa 14–685) and S2 (aa 686–1273) subunits. These subunits are in charge of receptor binding and membrane fusion, respectively. The N-terminal domain (NTD, aa 14–305) and the receptor-binding domain (RBD, aa 319–541) constitute the S1 subunit. On the other hand, the S2 subunit encompasses the fusion peptide (FP, aa 788–806), the heptapeptide repeat sequence 1 (HR1, aa 919–984), the heptapeptide repeat sequence 2 (HR2, aa 1163–1213), transmembrane domain (TM, aa 1213–1237) and cytoplasmic domain [6].

Spike protein is of particular interest for immunogenicity, therefore any changes in this gene may result in altered variants in terms of immune response or infectivity as novel Spike variants are increasingly recognized worldwide. A recently emerged variant in the United Kingdom raised the alarm, since this particular variant accumulated several mutations in an accelerated rate [7]. A remarkable example of Spike-mutants is the D614G variant, which emerged during the early phase of the pandemic and is now dominant in most parts of the world. Based on in vitro and also in vivo animal studies along with epidemiological data analysis, there is a growing concern for the modified phenotype of this variant coupled with elevated transmissibility and infectivity [8,9,10].

In the present study, we describe a recessive, deleterious Spike-protein mutant SARS-CoV-2 strain. We describe the co-infection with two different strains in a single patient, including the deleterious variant. We performed in silico protein analysis with the Spike RBD region, which includes the deletion. These results may support the general understanding of natural genomic processes of SARS-CoV-2. The complete genomic sequence of this variant may also facilitate future research activities where natural attenuated strains are essential.

## 2. Materials and Methods

### 2.1. Sample Collection, Andsequencing

Prior to the Nanopore sequencing (Oxford Nanopore Technologies, Oxford, UK), oropharyngeal swab were directly collected into viral transport medium (from the second and third timepoint 30 March 2020, 6 April 2020) and were stored at −80 °C. 200 µL of sample were used for nucleic acid extraction with the usage of Direct-zol RNA Miniprep (Zymo Research, Irvine, CA, USA) following the manufacturers recommendation. The RNA preparation for Nanopore sequencing were carried out by the ARTIC version 2 protocol with nCoV-2019 V3 primer set [11,12]. The sequencing of the amplicon libraries were performed MinION flow cell version 9.4.1 (Oxford Nanopore Technologies, Oxford, UK). The generated sequence data were processed by the ARTIC bioinformatic pipeline with minimum coverage cutoff value, which was 20 × [13].

In order to verify the deletion, we conducted an end-point PCR with the 76 amplicon primers of ARTIC nCoV-2019 V3 sequencing primer set [12] (nCoV-2019_76_LEFT: 5′- AGGGCAAACTGGAAAGATTGCT -3′, nCoV-2019_76_RIGHT 5′- ACACCTGTGCCTGTTAAACCAT-3′) which embrace the questionable genomic region. PCR reaction was performed with Q5 (New England Biolabs) with the following cycling conditions: 98 °C for 30 s then 40 cycles of denaturation at 98 °C for 15 s, annealing at 61 °C for 30 s, elongation at 72 °C for 1 min, and final elongation at 72 °C for 5 min. We used 4150 Tapestation system (Agilent) with High Sensitivity DNA ScreenTape for quality check. The generated amplicons were separated with gelelectrophoresis on 2% SeaKem LE (Lonza) agarose gel. The dividual bands were purified with Monarch DNA Gel Extraction Kit (NEB) and bidirectionally sequenced on ABI Prism 310 genetic analyzer platform (Applied Biosystems) with the BigDye Terminator v1.1 CycleSequencing Kit (Applied Biosystems, Foster City, CA, USA).

**Figure 1:**
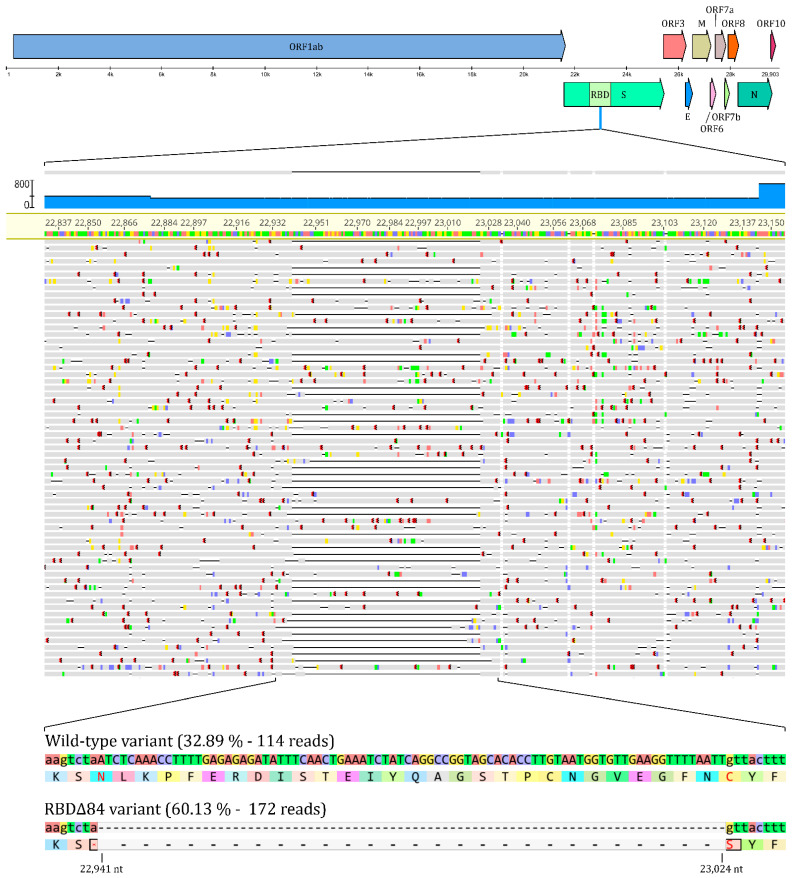
Representation of the deletion and its position within the SARS-CoV-2 genome, along with raw NGS sequencing data of the region of interest. The figure highlights the deleterious region and provides the quantitative ratio of the two variants during co-infection. Wild-type variant refers to the non-deleterious, whilst RBDΔ84 to the deletOrious variant. Visualization was partially performed in Geneious Prime 2020 software.

Visualization of Figure 1 and data management of raw sequence reads was performed in Geneious Prime 2020 software.

### 2.2. Clinical History and Other Details

A 59-year-old female patient without any known underlying disease or risk factors (i.e., smoking) tested positive for SARS-CoV-2 on 20 March 2020. She was working in Tirol, Austria till 15 March 2020 and travelled to Hungary on 17 March 2020. Given her travelling history, she had to be quarantined at home for 2 weeks. Her symptoms and PCR tests are summarized in Table 1. The first negative PCR test was obtained on 16 April, 2020. The loss of smell persisted for another month and weak numbness of the hands has remained as a residual symptom.

### 2.3. In Vitro Isolation

We used Vero E6 kidney cells (ATCC^®^CRL-1586™) for isolation which were previously reported to be highly susceptible to SARS-CoV-2 infection [14]. The cells were maintained in DMEM (Lonza, Switzerland) supplemented with 1% Penicillin-Streptomycin (Lonza, Switzerland) and 10% Fetal Bovine Serum (Biosera, Nuaillé, France) at 37 °C with 5% CO_2_ until 70% confluency in a T25 flask. For inoculation we used 200 µL of sample completed with 800 µl of DMEM. Cells were incubated for one hour at 37 °C with 5% CO_2_. After incubation, we replaced the inoculum with fresh DMEM (Lonza, Switzerland) supplemented with 1% Penicillin-Streptomycin (Lonza, Switzerland) and 2% Fetal Bovine Serum (Biosera, Nuaillé, France) and cells were monitored for cytopathogenic effect. All manipulations of infective virus isolate were conducted at the BSL-4 laboratory of the Szentágothai Research Centre, National Laboratory of Virology.

### 2.4. In Silico Protein Analysis

**Figure 2:**
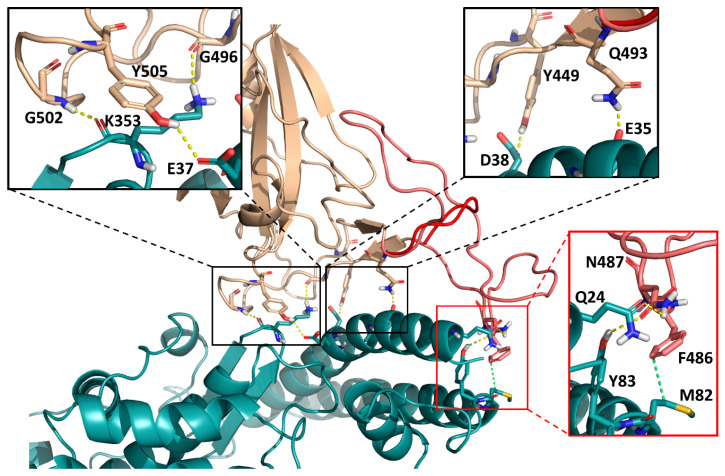
Interface of the ACE2 receptor (light blue) with the SARS-CoV-2 Spike protein receptor-binding domain (tan), visualized based on PDB structure 6M17. Amino acids that were identified as part of the three interaction segments are included as sticks and highlighted in the three insets. In the modeled RBDΔ84 mutant, an extended loop (amino acids 460–488, light red/salmon) is replaced by a compact turn (dark red, average structure of the MD simulation), removing the C-terminal interaction segment (red inset) and resulting in a significantly smaller contact surface with the ACE2 receptor (779 ± 31 Å2 for RBDΔ84 vs. 921 ± 3 Å2 for the wild-type protein). This, in turn, should result in weakened ACE2 binding.

The homology model of the RBD^Δ84^ mutant was prepared with Prime [15], based on the structure of the wild-type protein [16]. We have applied the knowledge-based modeling algorithm of Prime and kept the best five resulting homology models for each monomer (chain E and chain F of PDB structure 6M17). Despite the length of the deleted loop, modeling requires a relatively small modification of the conformation of the remaining loop elements, since the two end-points (N460 and C488) are relatively close to each other. The resulting models were refined with the Protein Preparation Wizard of Schrödinger, by optimizing their H-bond networks (PROPKA) and subjecting them to a restrained minimization (with heavy atom movements restricted to and RMSD value of 0.3 Å) [17]. Protein-protein contact surfaces were calculated with Schrödinger Maestro for the ACE2 residues whose distances from the RBD are at most 3Å (averages and standard deviations were calculated for two wild-type structures and ten refined RBD^Δ84^ homology models). To explore the conformational space of the shortened loop of the RBDΔ84 mutant, the best homology model (according to the Prime energy) was subjected to 50 ns of equilibrium MD simulations with Desmond [18] (five simulations with different initial velocities, 10 ns each), with large harmonic restraints (100 kcal/mol) on the positions of all backbone atoms, except for the residues of the shortened loop (positions 455–491, nine residues in total). The structure of the ACE2- RBDΔ84 complex was placed in an orthorhombic box, solvated in TIP3P water [19], and neutralized by adding sixteen sodium ions. The salt concentration was adjusted to 0.15 M by the addition of further 95 sodium and 95 chloride ions. The terminal residues of the proteins were capped with acetyl and N-methyl groups, respectively. The system was equilibrated with the default protocol of Desmond, and submitted to five independent, 10 ns-long production runs (with different initial velocities) with the OPLS3 [20] force field, in the NPT ensemble at 310 K, using a Nosé-Hoover thermostat [21,22] and a Martyna-Tobias-Klein barostat [23]. The trajectories were merged and clustered with the affinity propagation algorithm [24], and the representative structure of the largest cluster was included in Figure 2 as the average structure of the simulation. Figure 2 was rendered with Pymol [25].

## 3. Results

### 3.1. Sequence Analysis

A SARS-CoV-2 PCR positive sample from a 59 years old female patient was subjected to Nanopore sequencing, using the ARTIC protocol as a part of a molecular epidemiological surveillance program in Hungary. For the data analysis we used the ARTIC bioinformatic pipeline [13]. 32,584 reads were mapped to the reference genome (Accesion number: MN908947.3) which resulted in 469,5-fold mean coverage (min: 34, max: 1919) across the genome. The generated consense sequence contained 5 mutations at position 241(C -> T), 3037(C -> T), 14,408(C -> T), 23,403(A -> G), 24,862(A -> G) across the genome and a 84 base pair long deletion between position 22,941 and 23,024.

Amplicon number 76 of the ARTIC library preparation setup was affected by this deletion so we filtered and extracted these reads. This deletion had 286 X coverage and 60.13% (172) of the reads contained the deletion while the remaining 39.87% (114) were identical with the wild type reference genome, suggesting a co-infection with two variants. Details are summarized in Figure 1.

### 3.2. In Vitro Isolation

Isolation efforts on VeroE6 cell line resulted with no success. We were unable to establish an infectious isolate from the available samples of this strain.

### 3.3. Protein Modelling Results

We have used the online translator tool of ExPASy to translate the DNA sequence to an amino acid sequence [26]. From the three possible solutions (starting the reading frame from either the first, second, or third nucleotide), the third solution perfectly corresponds to positions 461–487 of the amino acid sequence of the RBD (Uniprot entry P0DTC2) [27], highlighted in bold: 

N**LKPFERDISTEIYQAGSTPCNGVEGFN**C

However, since the reading frame starts from the third nucleotide, the deletion also affects the two neighboring amino acids of the sequence (N460 and C488 coded by the aat and tgt codons, respectively). Consequently, amino acids corresponding to a total of 87 base pairs are affected (see above, full sequence). The deletion leaves the residual DNA sequence agt, to be translated into a serine residue. Therefore, the overall effect of the RBDΔ84 mutation is the replacement of the above 29 amino acid long sequence with a single serine residue.

Next, we have checked the recently published structure of the Spike RBD-ACE2 complex for an assessment of the structural effects of this mutation [16]. Also, we have produced homology models of the RBDΔ84 mutant (based on the wild-type structure) for a basis of comparison. To explore the conformational space of the shortened loop of the RBDΔ84 mutant, the best model was subjected to 50 ns of equilibrium MD simulations, with restraints on the backbone atom positions of the rest of the complex. Figure 2 shows the interface of SARS-CoV-2-RBD and ACE2, which entails the α1 helix of the ACE2 peptidase domain and an extended loop region of the RBD that includes the deleted sequence. The RBD arches over the α1 helix in a bridge-like structure with three main interaction segments [16]. The RBDΔ84 mutation essentially removes one of the interacting segments (as visualized by the average structure from the MD simulations in Figure 2), resulting in a significantly smaller contact surface with the ACE2 receptor (779 ± 31 Å2 for RBDΔ84 vs. 921 ± 3 Å2 for the wild-type protein). This is in line with the weakened ACE2 binding, as proposed.

The RBDΔ84 mutant lacks the residues Q474 and F486, forming key interactions in the RBD-ACE2 complex [16]. Furthermore, a recent full single-point mutational scan of the RBD [28] revealed that the Δ84 region contains a number of positions (L461, D467, Y473, C480, N487 and C488) whose mutations significantly deteriorate ACE2 binding. Based on these data it is reasonable to assert that the much larger perturbation observed in the RBDΔ84 mutant does have a deteriorating effect.

## 4. Discussion

Viral genomic surveillance has several advantages during an outbreak situation from direct assistance for public health decisions to the identification whether nucleotide changes in the viral genome may affect diagnostic of therapeutic practices [7,29,30]. Regarding RNA virus evolution, single nucleotide polymorphism and point mutations frequently occur. In case of coronaviruses, the most significant evolutionary driving force is recombination, which may result in insertions and deletions in the viral genome [4].

There is an increasing number of studies in association with the emergence of novel mutations within the SARS-CoV-2 genome. Compared to reports about mutations with amino-acid changes, there are few studies related to deleterious changes in the genome. Interestingly, the conserved genes of ORF3a and ORF7a were found to be affected by deletions. These genes regulate a wide range of functions during infection, such as activation of chemokine production, RNS silencing suppression, NLRP3 inflammasome activation, etc. [30,31,32,33]. Such deletion variants are hypothesized to manage milder disease manifestation in patients, however, to support this theory extensive investigations are necessary [34]. Other genomic regions were also reported with selective mutations, such as the ORF8 [34,35] and an additional twelve deletion site was discovered apart from ORF8, Spike and ORF7a [36].

Spike mutations are of special interest due to their possible role in the emergence of variants with modified antigenicity. This altered antigenicity may bypass vaccine effectiveness, monoclonal therapeutic options, and several others. A recent study revealed a 0.75% prevalence for Spike deleterious variants by analyzing 146,795 genome sequences. These deletions were mostly positioned within the N-terminal domain [37]. There are several identified Spike mutants rapidly spreading, such as the D614G which is now considered as a prevalent SARS-CoV-2 variant all around the world [38]. Sequential rounds of evolution in the context of an outbreak situation with an emerging virus was previously reported in case of SARS-CoV and during the West African Ebola outbreak. It is considered as an adaptation to the new human host and may lead to significant increase in the prevalence of certain variants [39,40].

Dominant mutations may drive the main scenario of an outbreak [7,10], but recessive mutations are also present, although the identification of the latter is highly challenging. The general understanding of SARS-CoV-2 evolution during the current pandemic situation may reveal future scenarios and can facilitate therapeutic research directions and strategies.

We identified a major deletion in the RBD of the Spike protein and verified an altered receptor binding capacity via in silico methods. The variant presented in this study lacks a major part of the RBD along with several important AA positions for ACE2 binding. We therefore hypothesized a weaker receptor binding capacity. The occurrence of such a mutation in a natural infection and the recessive nature of this deleterious variant revealed a scenario for evolutionary adaptation of the virus within a single host. The identification of this strain may be of high interest in future studies, involving attenuated strains and thereby it may facilitate therapeutic advances.

A limitation of our study is the lack of infectious isolate, since the virus isolation efforts failed to retrive infectious isolate in vitro. It is possibly due to the condition of the sample after multiple freeze-thaw cycles or even due to the lower specificity to VeroE6 cell line of this particular deleterious strain. Reverse genetics may be used in future studies for in vitro experiments with this deleterious variant to verify in silico data and to examine in vitro characteristics [41].

## 5. Conclusions

The most important take-home message of our study is that we need more studies to understand main genomic-evolutionary mechanisms of circulating SARS-CoV-2 viruses regarding their most powerful evolutionary tool, the recombination. Sequencing efforts therefore need to be focused on the surveillance of emerging recombinant variants, not only tracking nucleotide changes in the genome. Here, we present a naturally occurring recessive RBD deleterious SARS-CoV-2 variant. Strains with altered receptor-binding capacity may be of special interest in future studies in relation to vaccine development, therapeutic options or simply for the general understanding of evolutionary mechanisms regarding the COVID-19 pandemic.

## Figures and Tables

**Table 1:** Summary of the clinical history for the patient.

Date	PCR Diagnostic Result	Symptoms and Test
18 March 2020	n/a	Tiredness, headache, fever (38.3 °C)
19 March 2020	n/a	Loss of taste and smell, sore throat, fever
20 March 2020	**Positive**	Tiredness, limb weakness, subfebrility (37.5 °C), Charlson Comorbidity index (CCI): 1
22 March 2020	n/a	severe numbness in the limbs and tongue
30 March 2020	**Positive**	asymptomatic
6 April 2020	**Positive**	asymptomatic
16 April 2020	Negative	asymptomatic

## Data Availability

Data is contained within the article
